# Probing Interkingdom Signaling Molecules via Liquid Extraction Surface Analysis–Mass Spectrometry

**DOI:** 10.1021/acs.analchem.2c05703

**Published:** 2023-03-07

**Authors:** Shaun N. Robertson, Fadi Soukarieh, Thomas M. White, Miguel Camara, Manuel Romero, Rian L. Griffiths

**Affiliations:** † U.K. National Biofilm Innovation Centre (NBIC), Biodiscovery Institute, School of Life Sciences, Faculty of Health and Medical Sciences, University of Nottingham, NG7 2RD Nottingham, U.K.; ‡ Faculty of Science, School of Pharmacy, University of Nottingham, NG7 2RD Nottingham, U.K.; § Department of Microbiology and Parasitology, Faculty of Biology-CIBUS, Universidade de Santiago de Compostela, 15782 Santiago de Compostela, Spain

## Abstract

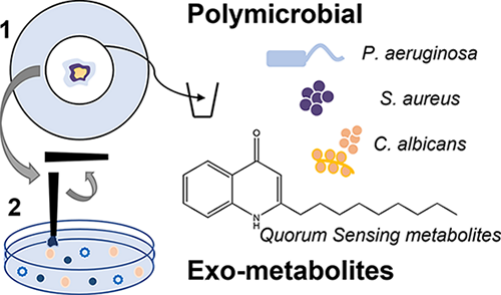

Previously, metabolites diffused or secreted from microbial samples have been analyzed via liquid chromatography–mass spectrometry (LC–MS) approaches following lengthy extraction protocols. Here, we present a model system for growing biofilms on discs before utilizing rapid and direct surface sampling MS, namely, liquid extraction surface analysis, to study the microbial exometabolome. One of the benefits of this approach is its surface-specific nature, enabling mimicking biofilm formation in a way that the study of planktonic liquid cultures cannot imitate. Even though *Pseudomonas aeruginosa* (*P. aeruginosa*), *Staphylococcus aureus (S. aureus),* and *Candida albicans* (*C. albicans*) have been studied previously in isolation, very few studies consider the complexity of the interplay between these pathogens, which are commonly combined causative agents of infection. Our model system provides a route to investigate changes in the exometabolome, such as metabolites that become circulatory in the presence of multiple pathogens. Our results agree with previous reports showing that 2-alkyl-4(1*H*)-quinolone signal molecules produced by *P. aeruginosa* are important markers of infection and suggest that methods for monitoring levels of 2-heptyl-4-hydroxyquinoline and 2,4-dihydroxyquinoline, as well as pyocyanin, could be beneficial in the determination of causative agents in interkingdom infection including *P. aeruginosa*. Furthermore, studying changes in exometabolome metabolites between *pqs* quorum sensing antagonists in treated and nontreated samples suggests suppression of phenazine production by *P. aeruginosa*. Hence, our model provides a rapid analytical approach to gaining a mechanistic understanding of bacterial signaling.

## Introduction

Polymicrobial biofilms are common throughout healthcare, industrial, and environmental settings. These surface-associated or aggregative microbial communities are central to the challenge of antimicrobial resistance due to the overproduction of extracellular polymeric substances that can entrap and limit the diffusion of certain antibiotics,^1^ and the induction of physiological changes that lead to antimicrobial tolerance in cells.^2^ It is estimated that 65% of bacterial infections are biofilm-associated^3^ and while underestimated, *Candida albicans* (*C. albicans*) biofilm infections are responsible for an estimated >400,000 life-threatening infections a year worldwide with a mortality rate of 46–75%.^4^ In these communities, microbes convey their presence by producing small diffusible signal molecules known as autoinducers, which can then be detected and responded to in a population-coordinated manner. This form of microbial intercellular communication is called quorum sensing (QS)^5^ and regulates physiological processes in these communities and, importantly, has been shown to control biofilm formation.^6^ Remarkably, QS signaling molecules have previously been shown to act as biomarkers of lung infection via LC–MS of plasma from cystic fibrosis patients.^7^ Hence, these molecules could represent important circulatory indicators of infection that could be tested noninvasively.

Given that most biofilm-centered infections are polymicrobial,^8,9^ the possibility of interspecies microbial communication can also occur and QS is thought to play a vital role in interspecies interactions ranging from commensalism to antagonism.^8^ For instance, interkingdom QS signaling between the bacterium *Pseudomonas aeruginosa* (*P*. *aeruginosa*)and fungi *C. albicans* species has been described^9^ as mediated via the production of 2-alkyl-4(1*H*)-quinolone (AQ) signal molecules and farnesol, respectively. Moreover, these species are often present alongside *Staphylococcus aureus (S. aureus)* in healthcare settings^10,11^ and it is well documented that *P. aeruginosa* displays severe competition against *S. aureus* in vivo. For example, in wound infections or cystic fibrosis lung, extracellular factors produced by *P. aeruginosa* have been shown to subjugate *S. aureus* to persist as small colony variants.^12^ One of such factors is the AQ molecule 2-heptyl-4-hydroxyquinoline N-oxide (HQNO), a cytochrome inhibitor released by *P. aeruginosa*. In turn, *P. aeruginosa* can sense extracellular products secreted by *S. aureus* such as the exopolymer *N*-acetyl glucosamine, and in response, increase the production of virulence factors and antimicrobials.^5,12,13^ Similarly, farnesol, farnesoic acid,^14^ and various amino acid-derived alcohol^15−17^ molecules secreted by *C. albicans* have been shown to play an important role in biofilm formation^18,19^ and are currently under-investigated in the context of polymicrobial biofilms.

Mass spectrometry (MS) offers a route to the untargeted analysis of multiple species without requiring chemical tagging/modification. Surface sampling approaches offer the opportunity to directly probe solid biological samples such as tissue sections, blood spot cards, and bacterial biofilms. Many microbial species have been analyzed via direct surface sampling MS approaches. The aims of these studies vary from bacterial phenotyping to drug screening and understanding bacterial biofilms. Various biomolecules have been studied via different approaches; antimicrobials, bacterial QS signaling molecules, metabolites, and rhamnolipids have been studied via matrix-assisted laser desorption ionization (MALDI) ionization MS,^20−22^ and secondary ionization mass spectrometry (SIMS).^22−28^ Phospholipid analysis is widely reported via rapid evaporative ionization mass spectrometry (REIMS)^29^ and desorption electrospray ionization (DESI) approaches, and lipids and intact proteins have been studied by liquid extraction surface analysis–mass spectrometry (LESA-MS).^30−32^ Many REIMS studies focus on speciation using mathematical algorithms to understand profiles of, e.g., bacterial strains including *P. aeruginosa*(29,33) and fungi such as the *Candida* genus,^33,34^ without identifying specific analytes. Previously, proteins involved in the interaction between *P. aeruginosa* and *S. aureus* have been investigated via bottom-up proteomics/MALDI-MS imaging and correlated to metal ions (involved in protein interactions) by laser ablation-inductively coupled plasma MS imaging.^35^ Differences in the lipid and metabolite profiles (including heme, porphyrin, and antibiotic compounds) of *Shewanella oneidensis* and *Bacillus subtilis* as single species and mixed biofilms have been investigated via nano-DESI.^36^

LESA-MS is a surface sampling approach that utilizes solvent extraction into a robotically operated pipette tip prior to electrospray ionization. LESA-MS offers soft ionization and rapid analysis time (a few minutes) with the additional benefit of long spray times, particularly useful for structural identification (MSMS) experiments. Previously, LESA-MS of microbial colonies including *P. aeruginosa*, *S. aureus,* and ESKAPE pathogens has been described for the analysis of intact proteins from a range of mono-microbial systems.^30,31,37,38^ More recently, LESA-MS has been used for the direct bacterial analysis of lipids from *Mycobacterium*(30−32,38) and has been suggested as a potential alternative to MALDI-MS in speciation of clinical isolates. Here, we describe for the first time exometabolite analysis specifically, and from mono- and *poly*-microbial biofilms.

This study seeks to investigate *P. aeruginosa* QS signaling molecules diffused and/or secreted from single and polymicrobial species biofilms using a novel sample preparation method specifically designed to understand the bacterial exometabolome. LESA-MS is described for the first time for targeted analysis of secreted QS molecules; in the agar underneath a biofilm grown on a disc. Differences in QS were determined in the presence of a microbial competitor (namely, *S. aureus* or *C. albicans*), and in the presence of both competitors using the LESA-MS platform. Our model allowed the detection of the AQ signal 2-heptyl-4-quinolone (HHQ), previously identified as a diagnostic marker of *P. aeruginosa* infection, and suggested the utility of our approach. Our results also suggest that methods for monitoring levels of other AQs such as 2,4-dihydroxyquinoline (DHQ), as well as pyocyanin, could be beneficial in the determination of causative agents in interkingdom biofilm communities. Furthermore, the effect of antibiotic treatment and/or inhibition of the AQ-based communication system in *P. aeruginosa* was examined and our study shows that, in agreement with previous reports, AQ inhibitors can suppress phenazine biosynthesis in biofilm communities including this bacterial pathogen. Therefore, our model provides a rapid analytical approach to gaining a mechanistic understanding of QS processes in the context of polymicrobial biofilms.

## Experimental Section

### Materials and Methods

#### Strain and Colony Biofilm Culture Conditions

*S. aureus* SH1000 and *P. aeruginosa* PAO1-L strains were routinely grown on the lysogeny broth (LB, Oxoid, Cambridge, UK) agar. *C. albicans* SC5314 was routinely grown in the Sabouraud dextrose (SAB, Oxoid, Cambridge, UK) agar. Bacterial and fungi plates were incubated at 37 and 30 °C, respectively. UVC-sterilized polycarbonate (PC) discs (13 mm diameter) on wells of 6-well plates filled with 5 mL of media. The stock inoculum (10 μL, added on top of one another for polymicrobial combinations) was pipetted to the center of the PC disc. Plates were then transferred to a static incubator and incubated at 37 °C for 24 h to allow colony biofilm growth. For treatment with the QS inhibitors (QSIs), SEN19 and SEN89 compounds were supplemented at 10 μM in the final stock inoculum of PAO1-L. Treatment of 18 h PAO1-L colony biofilms with ciprofloxacin was performed by adding 20 μL of 64 μg/mL in H_2_O pipetted gently on top of the preformed colony biofilm. At the endpoint, PC discs with attached colony biofilms were aseptically removed and agar plugs were used for QS signal detection. Triplicate biological and technical repeats were conducted for all experiments presented. Further details, including CFU counting details, can be found in the Supplemental Information.

#### LESA Sampling

LESA was carried out using the Triversa Nanomate (AdvionBiosciences, Ithaca, NY, USA). The extraction/ionization solvent was 1:1 methanol/water. During extraction, 5 μL of solvent was aspirated from the solvent well, before sampling the agar with 2 μL of this solvent for 5 s twice (1 mix). Finally, 2.5 μL of the sampling solvent was re-aspirated and infused into the mass spectrometer at a gas pressure of 0.3 psi and a potential of 2.0 kV.

#### Mass Spectrometry

Experiments were performed on a Thermo Fisher Orbitrap Q-Exactive mass spectrometer. Mass spectra were recorded in full scan mode at a resolution of 140,000 at *m/z* 400 in the *m/z* range of 50–750 in positive ionization mode. The AGC target was 1 × 10^6^ charges with a maximum injection time of 500 ms. Each scan consisted of 1 microscan. MSMS details can be found in the Supplemental Information. Data were recorded for up to 1 min and analyzed using Thermo Xcalibur version 4.2.28.14 software. MSMS spectra were manually interpreted.

## Results and Discussion

### Alkyl-quinolone (AQ) Quorum Sensing (QS) Signaling Molecules Diffused from and Virulence Factors Secreted by *P. aeruginosa* Biofilms Detected by LESA-MS

To assess whether LESA-MS could be used to study the release of small organic molecules from microbial biofilms, we studied the presence of QS molecules, which are most likely passively diffused, and pyocyanin secreted by 24 h *P. aeruginosa* PAO1-L monospecies biofilms. Details regarding specific QS pathways and molecules analyzed are provided in the Supplemental Information.

**Figure 1:**
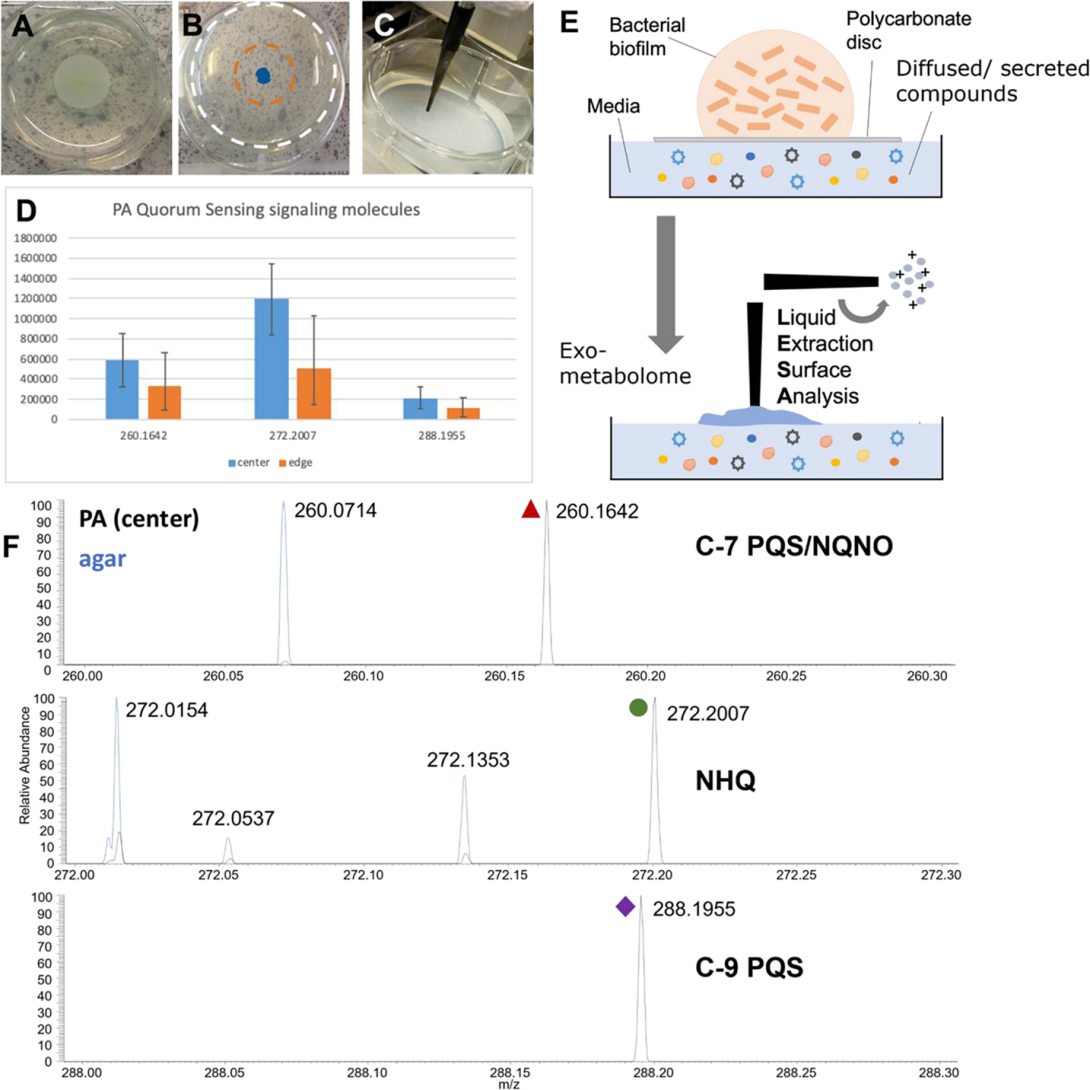
Exometabolome analysis of QS molecules from *P. aeruginosa* PAO1-L biofilms. Photos of (A) biofilm grown on PC disc, (B) agar sampled after removal of the disc with locations indicated (blue = center, orange = edge), and (C) LESA sampling. (D) Bar charts showing decreasing signal intensities of selected QS molecules from the center and the edge of where the biofilm was grown on the PC disc. (E) Schematic of the sample preparation for exometabolome analysis and LESA sampling. (F) Spectra showing LESA-MS of agar background (blue) and selected QS molecules detected in the exometabolome of *P. aeruginosa* biofilms.

**Figure 2:**
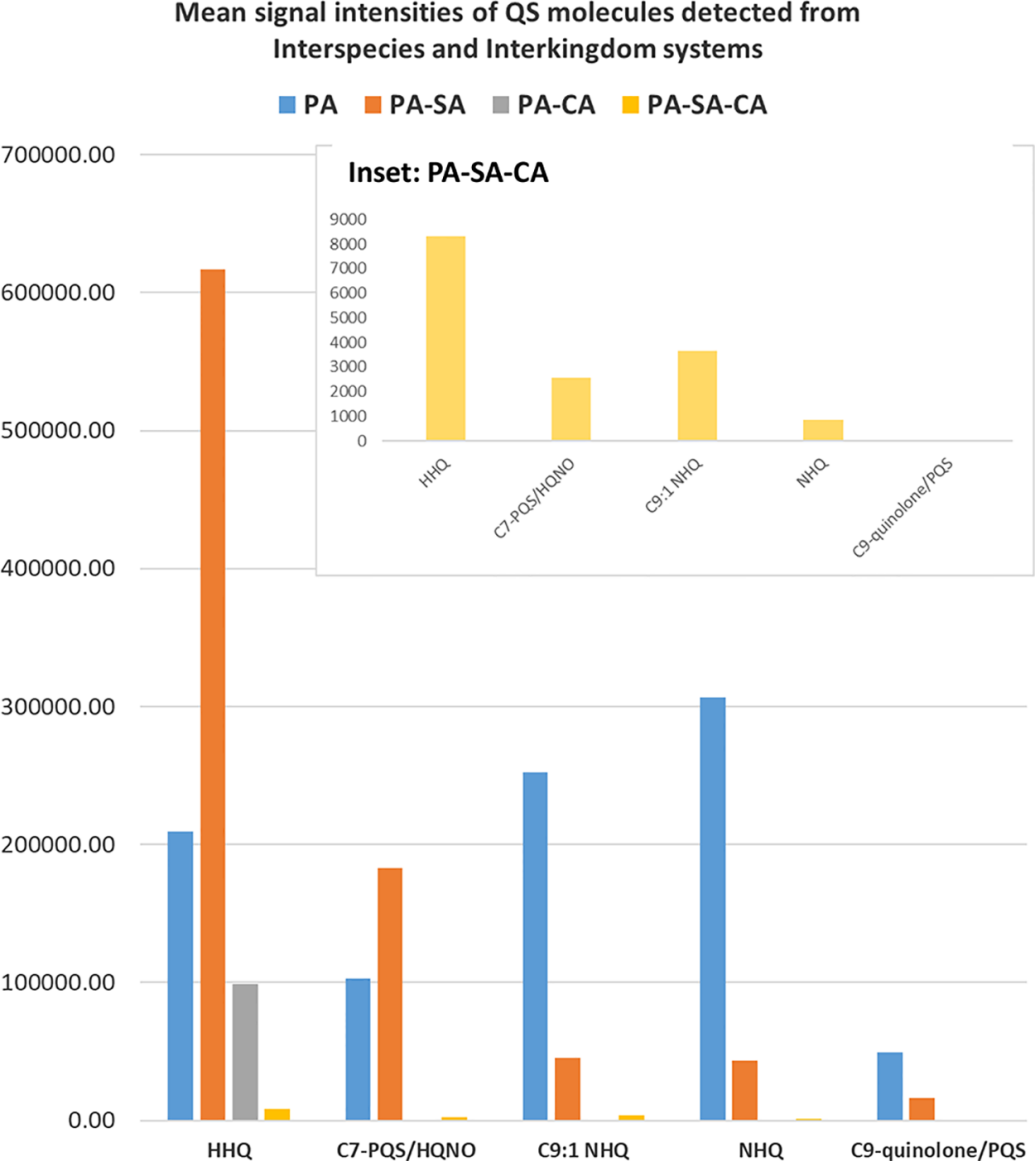
Bar chart showing the mean signal intensity of QS molecules detected via LESA-MS in the exometabolome analysis of interspecies biofilms. Mean values for PA–SA–CA, which were lower than other biofilms are shown in the inset.

To allow cell-free metabolite extraction from agar substrates, biofilms were grown on 0.2 μm pore PC discs placed on the agar medium to enable the aseptic removal of the microbial cells prior to LESA-MS analysis (Figure 1). The following *P. aeruginosa* signals were detected from the media: HQNO, C9:1-PQS, C9-PQS, C9 quinolone, C11 quinolone, and HHQ, see Figures 1 and 2. These fall into three classes of AQ QS molecules: HHQ-derived compounds [*m/z* 244.1696 (HHQ), 270.1849 and 272.2007], PQS-derived compounds (*m/z* 260.1642 and 288.1955), and HQNO-derived compounds [*m/z* 260.1642 (HQNO) and 288.1955], see Table 1. Note that some of these compounds are isomers of one another, therefore MSMS is required for elucidation. However, N-acyl homoserine lactone (AHL) signal molecules were not detected in the conditions tested. The virulence factor pyocyanin ([M + H]^+^*m/z* 211.0863) was detected and secreted from PAO1-L biofilms, suggesting that our novel sample preparation methodology allows detection of various diffused and/or secreted metabolites external to the biofilm. LESA-MS was previously described for analyzing intact bacterial proteins and lipids.^32−34^ However, to our knowledge, QS autoinducers have not previously been reported using this approach.

**Table 1:** Metabolites Detected in the Exometabolome of Interspecies and Interkingdom Biofilms of *P. aeruginosa* (PA), *S. aureus* (SA), and *C. albicans* (CA), and in the Exometabolome of Ciprofloxacin (CIP) Drug and/or (SEN019 or SEN089) Inhibitor-Treated PA Biofilms^a^

detected *m/z*	accurate *m/z*	ppm	chemical formula	assignment	PA	PA–SA	PA–CA	PA–SA–CA	PA–CIP	PA–SEN019	PA–SEN019-CIP	PA–SEN089
244.1696	244.1701	2.2	C_16_H_22_NO	HHQ	*	*	*	*	*	*	-	-
260.1642	260.1651	3.5	C_16_H_22_NO_2_	HQNO/C7-PQS	*	*	-	*	*	*	-	*
270.1849	270.1858	3.3	C_18_H_24_NO	C9:1 quinolone	*	*	-	*	*	*	-	-
272.2007	272.2014	2.6	C_18_H_26_NO	NHQ	*	*	-	*	*	*	-	-
288.1955	288.1964	3.1	C_18_H_26_NO_2_	C9 PQS	*	*	-	-	*	*	-	-
300.232	300.2327	2.3	C_20_H_30_NO	C11 quinolone	*	*	*	-	-	*	-	-
211.0863	211.0871	3.8	C_13_H_11_N_2_O	pyocyanin	*	*	*	-	*	-	-	-
188.1642	188.1651	4.8	C_10_H_10_N_2_O_2_	cis-2-decenoic acid (CDA)	-	-	-	*	-	*	-	-
162.0548	162.0555	4.3	C_9_H_8_NO_2_	dihydroxyquinolone (DHQ)	-	-	-	*	-	*	-	-

a Key: - = not detected reliably, * = detected reliably across repeats.

QS molecules have previously been analyzed using, e.g., SIMS, from biofilms ranging from 7 to 72 h of growth.^22,27,28^ AQs with C9 alkyl chains were shown to be highly abundant in early 7 h biofilms of *P. aeruginosa* grown on silicon tiles.^28^ Similar molecules have been reported via MALDI imaging of 12 h biofilms.^21,39^ These QS signaling molecules have also been reported in mature (72 h) biofilms of *P. aeruginosa* grown on silicon wafer tiles.^40^ Here, we show that AQs derived from HHQ, PQS, and HQNO can be analyzed via LESA from biofilms grown for 24 h. There are numerous benefits to our approach. First, previous reports have studied biofilms directly; hence, it is not possible to distinguish between metabolites within the biofilm and those diffusing away from the biofilm. Here, we present a new sample format specifically designed to analyze bacterial biofilm exometabolites that also has the advantage of understanding the effect of distance on microbial QS, which could be important in determining diagnostic markers of infection/severity. Comparison of the exometabolites detected in the media at the site of the colony growth (blue, Figure 1B), and then around the outer edge of the PC disc (orange, Figure 1B, and further away (white, Figure 1C), shows decreasing signal intensities of the following signals: *m/z* 260.1642 (HQNO), *m/z* 272.2007 (C9-quinolone/2-nonyl-4(1*H*)-quinolone (NHQ)) and *m/z* 288.1955 C9-PQS/NQNO, see Figure 1A,B,D. On a practical level, our approach allows the analysis of biomolecules diffused/secreted from bacterial biofilms without needing a dedicated instrument in the microbial laboratory, subject to the appropriate biological safety measures.

Our LESA experiments benefit from coupling to a high-resolution (Orbitrap) mass analyzer offering the advantage of accurate mass (within 5 ppm), see Table 1. Previous surface-sampling MS studies into QS directly from biofilms have typically utilized either SIMS or MALDI, which are more commonly coupled to time-of-flight instrumentation that does not offer the same capabilities. Only recently has Orbitrap SIMS instrumentation become available,^41^ reporting the detection of 33 AQs and 6 AHLs in biofilms of *P. aeruginosa* cultured for 48 h.^42^ The differences with our results could arise due to differences in sampling time points (24 vs 48 h biofilms), sampling of the exometabolome rather than direct biofilm analysis, and/or the lower diffusion and stability of AHLs dispersing for the biofilms.

Our approach also benefits from MSMS capability, allowing structural characterization of detected molecules. Hybrid instruments with this capability have only recently been described in the SIMS community.^43^ MSMS experiments confirmed *m/z* 244.17 is HHQ, see the Supplemental Information and Figure S1. HHQ has recently been shown to have clinical diagnostic relevance in the early detection of infection from noninvasive biological fluids such as urine and breath condensate in critically ill patients with infections characterized by polymicrobial biofilms with bacterial and fungal contributors.^44^ HCD fragmentation of *m/z* 260.16 suggests this is a mixture of HQNO and PQS. Upon dissociation of *m/z* 272.20, the following product ions were detected: *m/z* 159.08, 172.07; however, there was no fragment at *m/z* 188.10 or 175.06 suggesting that this species is NHQ rather than C9-PQS. This was detected in relatively high abundance and has previously been shown to be an important biomarker of lung infection via LC–MS of plasma from cystic fibrosis patients.^7^ This demonstrates the potential of this approach as a model for the study of secreted biomolecules that enter the circulatory system and can inform QS molecules that could be monitored via noninvasive biological samples and are indicative of infection.

### Interspecies and Interkingdom Exometabolome Analysis

Using our LESA-MS approach, we studied the exometabolome of interspecies biofilms, including *P. aeruginosa*–*S. aureus* (PA–SA), and interkingdom biofilms of *P. aeruginosa*–*C. albicans* (PA–CA), and *P. aeruginosa*–*S. aureus*–*C. albicans* (PA–SA–CA) combinations. Colony biofilms of *P. aeruginosa* PAO1-L, *S. aureus* SH1000, and/or *C. albicans* SC5314 mixtures were co-cultured for 24 h, and the agar substrates were analyzed via LESA-MS. QS signals have previously been reported in interspecies interactions from 24 h biofilms grown at a 5 mm distance.^20^ Here, we report them from biofilm colonies grown with no spatial separation.

#### PA–SA Biofilms

Similar AQ molecules were observed in the exometabolome of PA–SA communities compared to PA biofilms, however, relative abundances were different, see Figure 2. HHQ was particularly abundant in PA–SA, with C7-PQS/HQNO also detected in higher abundance than in PA alone. In PA biofilms, the most abundant AQ detected was NHQ, followed by C9:1-quinolone/NHQ and then HHQ and HQNO/C7-PQS. Yet, in the PA–SA, HHQ was the most abundant, followed by C7-PQS/HQNO, with NHQ significantly decreased. This agrees with previous MALDI imaging studies of similar polymicrobial systems, which detected HHQ from *P. aeruginosa* (DK2-P2M24-2003 strain) in the presence of *S. aureus* (JE2).^20^ Our results also suggest that HQNO production from *P. aeruginosa* PAO1-L increases in the presence of *S. aureus* SH1000. Both HHQ and C9-PQS have been shown to repress *S. aureus* spreading.^45^ In these assays, there was no observed difference in the viable recovery of both microbial species when co-incubated at 24 h when compared to monospecies growth. Pyocyanin was detected in relatively high abundance via LESA-MS in PA–SA, a threefold increase in comparison to PA, see Figure S2. Pyocyanin is actively secreted from PA; therefore, it is a particularly useful biomarker to target, it is also toxic to SA, hence an increase in production is not unexpected. It is promising that our approach can determine a significant change in pyocyanin. Numerous attempts at modified pyocyanin assays have not yielded meaningful results, possibly owing to sensitivity limitations. Potentially, this highlights a benefit of our approach, although further validation is required.

#### PA–CA Biofilms

In the presence of *C. albicans* SC5314, HHQ was detected however, most AQ molecules were reliably suppressed (Figure 2), suggesting an important role of HHQ in *P. aeruginosa* survival in the presence of *C. albicans* or, contrary to SA competition, a potential disruption in the conversion of HHQ to PQS by CA. HHQ has previously been shown to affect *C. albicans* biofilm formation, and it is thought to play an essential role in PA–CA interkingdom interactions.^46^ Moreover, CFU counting showed a reduction in CA growth in the presence of PA with a 1–2 log difference in total cell counts. Inhibition of HQNO production suggests potential disruption to the PpqsL enzymatic activity in the PAO1-L pathway; however, this pathway is not disrupted in PA–SA. Pyocyanin was detected, although at lower abundance (∼sixfold decrease compared to PA biofilm yields) in the presence of CA. Future work will focus on validating reductions in AQs and pyocyanin as potential biomarkers of infection caused by PA in the presence of CA.

#### PA–SA–CA Biofilms

Despite HHQ and HQNO molecules being the most abundant AQs produced by PA in polymicrobial biofilms, including SA and CA (Figure 2), a significant reduction in these AQ yields was recorded (with no AQs detected in some repeats), suggesting competition from these could affect the PQS signaling system. Results indicate that, under the tested conditions, HHQ could be a good indicator of *P. aeruginosa* colonization, supporting the potential of this QS signal as a prospective early indicator of infection by this pathogen even in the context of polymicrobial infections. Interestingly, the AQ DHQ, contrary to single and dual-species biofilms including PA, was consistently detected in the PA–SA–CA community exometabolome (*m/z* 162.0548, Δppm 4.3). In contrast to HHQ and PQS, DHQ can be produced under low oxygen conditions,^47^ suggesting the reduction in HHQ and PQS yields and detection of DHQ could be explained by the formation of anaerobic niches in PA–SA–CA communities. Moreover, DHQ has been shown to act as a QS molecule to activate the response regulator PqsR for transcription of the *pqs* operon in *P. aeruginosa* in the absence of PQS and HHQ signaling and has been reported to alleviate the inhibition of PqsR by farnesol, albeit at a lower level compared with PQS.^47^ Cis-2-decenoic acid (CDA) was detected at *m/z* 188.1642 Δppm 4.8 in this triple system, and not in the other samples. Similarly to the PA–CA system, a significant decrease in pyocyanin was observed in PA–SA–CA compared to PA biofilms, which is in agreement with a reduction in the PQS system activity as it has been reported that phenazine production in *P. aeruginosa* is under the control of this QS regulatory system.^48^ Future work will focus on validating HHQ, HQNO, and DHQ presence and their potential use, together with pyocyanin, as biomarkers of polymicrobial infections including *P. aeruginosa*.

#### Summary

Overall, a range of alkyl-quinolones (AQs) was detected in the exometabolome of PA only. Particularly high levels of HHQ and pyocyanin in the exometabolome were indicative of PA in combination with SA causative agents. Low abundances of all AQs were detected when PA was co-cultured with CA, alongside a significant decrease in pyocyanin. When PA was co-cultured in the presence of both SA and CA, again many of the AQs that were detected in the PA-only system were suppressed, whereas DHQ and CDA were detected for the first time and therefore represent potentially informative ions alongside AHLs. When SA was co-cultured with CA, many ions that were detected were similar to those present in the exometabolome of the PA–SA–CA system, with the exception of CDA and DHQ. Our findings are summarized in Figure S3.

### Platform for Testing Therapeutic Strategies: *Pseudomonas aeruginosa* QS Inhibitors, Ciprofloxacin, and Adjunctive Strategies

**Figure 3:**
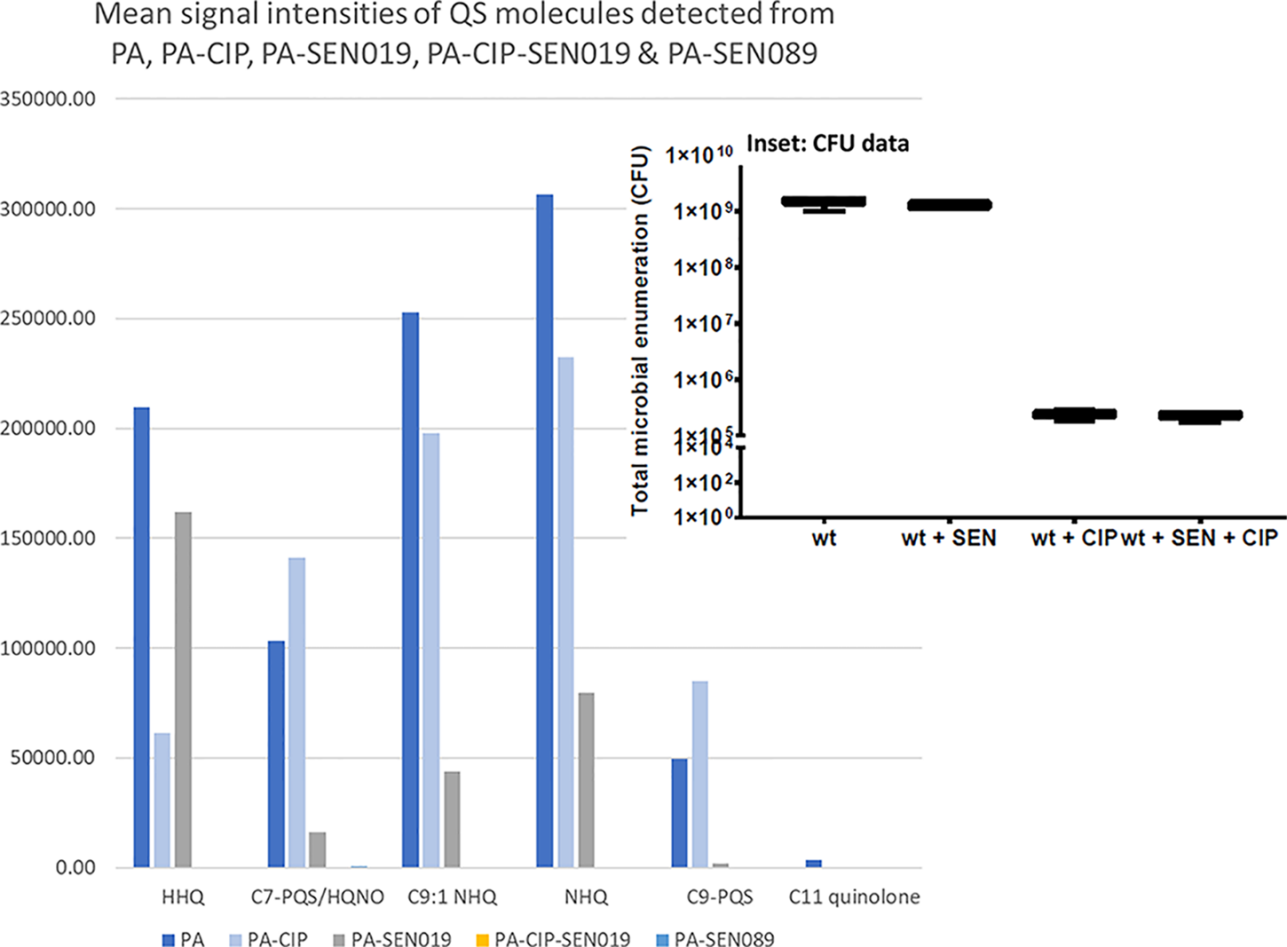
Bar chart showing the mean signal intensity of QS molecules detected via LESA-MS in the exometabolome analysis of treated PA biofilms. CFU measurements from PA, inhibitor (SEN019)-treated PA, and ciprofloxacin drug (CIP)-treated PA are shown in the inset.

Changes in QS molecules and virulence factor production could also potentially be used to monitor the effectiveness of therapeutic interventions. To study changes in these biomolecules upon treatment, biofilms of *P. aeruginosa* PAO1-L were cultured for 24 h in the presence of the antibiotic ciprofloxacin and/or the PqsR antagonists SEN019^49^ (a moderate inhibitor) and SEN089 (a potent inhibitor). These QSIs interfere with the binding of AQ QS signals to the PqsR response regulator leading to reduced activation of the *pqs* operon expression and therefore reduced biosynthesis of AQ signals and virulence factors in *P. aeruginosa*. Despite CFU counts showing a reduction in cell viability in PAO1-L biofilms treated with ciprofloxacin (∼4-log reduction), most AQ molecules detected in the exometabolome of untreated PAO1-L biofilms were found in communities treated with the antibiotic, e.g., HHQ (*m/z* 244.1696), C7-PQS/HQNO (*m/z* 260.1642), C9:1-quinolone (*m/z* 270.1849), and C9-quinolone/NHQ (*m/z* 272.2007). However, in this experiment, HHQ was significantly reduced in signal intensity across biological and technical repeats, and C7-PQS and C9-PQS were slightly elevated. In contrast, the *P. aeruginosa* virulence factor pyocyanin abundance was not significantly altered by ciprofloxacin treatment, see Figure 3.

In the presence of QSI SEN089 (IC_50_ = 67 nM,^46^), no AQ molecules were detected in the PAO1-L biofilm exometabolomes across all experimental biological and technical repeats, see Figure 3. This suggests that the presence of this inhibitor effectively disrupts the AQ biosynthetic pathway. Previously, QS molecules production was shown to decrease in planktonic cultures in the presence of various *pqs* inhibitors.^46,49^ Here, we show similar results from a sampling format specifically designed for the analysis of surface-associated microbial communities. This highlights one of the key benefits of our surface-based approach over alternative liquid-based approaches. Notably, pyocyanin secretion was also decreased in PAO1-L biofilms treated with this QSI, suggesting the suppression of *phzAB* expression in line with previous phenotypic analysis done with these QS antagonists.^46,49^ When PAO1-L biofilms were treated with the less potent QSI SEN19 (IC_50_ = 1 μM,^49^), AQ molecules could still be detected in the biofilm exo-metabolome albeit at a reduced yield. Similarly, to untreated biofilms, HHQ remained the most abundant AQ followed by NHQ, see Figure 3. In addition, pyocyanin displayed a secretion decrease after SEN19 treatment (Figure S3), again indicating suppression of *phzAB* expression. As expected from treatment with antivirulence compounds, CFU experiments showed no reduction in viable counts in the presence of both QSI inhibitors compared to untreated PAO1-L biofilms. CDA and DHQ were detected when PA was treated with the less potent inhibitor (SEN019), however, these were not detected upon treatment with a more potent inhibitor (SEN089).

Interestingly, biofilms cultured in the presence of SEN019 inhibitor and ciprofloxacin led to no detection of the common AQs present in PAO1-L exometabolome. Moreover, under combinatory treatment, no secreted pyocyanin could be detected, suggesting a global disruption of AQ-based QS networks and the potential of LESA-MS-based approaches for studying mechanisms of drug action of new anti-virulence treatments and adjunctive therapies. In contrast, CFU counts showed a similar decrease in biofilm cell viability (∼4-log reduction) compared to biofilms treated with ciprofloxacin only. These results indicate that the combination of these two drugs could contribute to a reduction in *P. aeruginosa* virulence but not to a further reduction in cell viability of treated biofilms under the conditions tested.

## Conclusions

This study demonstrates that the LESA-MS sampling format described here provides a suitable model for the analysis of the microbial exometabolome; with a range of clinically relevant metabolites detected in the studied microbial communities, including interspecies and interkingdom systems. Our approach has the added benefit of allowing microbiological cell culture in a laboratory with the appropriate safety measures for the specific pathogen, without the need for a dedicated instrument in the same lab. Furthermore, diffused or secreted compounds can be analyzed using our approach. HHQ has previously been indicated as an early diagnostic marker of infection in *P. aeruginosa*; our study agrees and therefore shows promise as a model. Additionally, our results suggest that methods for monitoring levels of AQs such as DHQ and pyocyanin could be beneficial in the determination of causative agents in interkingdom infection. Further investigation is required for validation; quantitative analysis of levels in circulatory biofluids via, e.g., LC–MS would be beneficial. Finally, we show how the LESA platform could provide a route to assessing the effect of drug treatments and adjunctive therapies for the future study of appropriate treatments for bacterial, interbacterial, and interkingdom systems using a bacteria-free platform.

We envision that our model will find use in rapidly investigating bacterial exometabolome analytes such as QS molecules from bacterial and interkingdom systems that will aid prediction of clinically relevant diagnostic metabolites of infection in bacterial, fungal, and interspecies/interkingdom systems that mimic the true complexity of chronic infection. This has relevance in the investigation and subsequent prediction of clinically relevant biomarkers that can be monitored noninvasively from biological fluids. A variety of interspecies and interkingdom systems could be studied in the future using this platform.

## Supporting Information Available

Expanded Experimental and Results sections, and Supplemental Figures 1–3 (PDF)
