# Supplementary material for: Probing Interkingdom Signaling Molecules via Liquid Extraction Surface Analysis–Mass Spectrometry
Source: Anal Chem. 2023 Mar 7;95(11):5079–86. doi: 10.1021/acs.analchem.2c05703 (PMC10034741; doi:10.1021/acs.analchem.2c05703)
Supplement: Supplementary file 1 — ac2c05703_si_001.pdf [file ac2c05703_si_001.pdf]

Supplemental information for Probing Inter-kingdom Signalling Molecules via LESA-MS

Shaun N Robertson[1], Fadi Soukarieh[1], Thomas M. White[2], Miguel Camara[1], Manuel Romero[1,3], Rian L. Griffiths[2]

[1]UK National Biofilm Innovation Centre (NBIC), Biodiscovery Institute, School of Life Sciences, Faculty of Health and Medical Sciences, University of Nottingham, NG7 2RD, Nottingham, United Kingdom

[2]School of Pharmacy, University of Nottingham, Faculty of Science, University of Nottingham, NG7 2RD, Nottingham, United Kingdom

[3]Department of Microbiology and Parasitology, Faculty of Biology-CIBUS, Universidade de Santiago de Compostela, 15782 Santiago de Compostela, Spain.

Keywords: Quorum sensing, Pseudomonas aeruginosa, Staphylococcus aureus, Candida albicans, polymicrobial biofilms, mass spectrometry, LESA-MS

Corresponding authors: rian.griffiths@nottingham.ac.uk and manuelromero.bernarez@usc.es

Contents

Supplemental information for Probing Inter-kingdom Signalling Molecules via LESA-MS 1

Experimental 2

Materials & Methods **2**

Results 4

Quorum Sensing Molecules **4**

MSMS **4**

Supplemental Figures 5

Supplemental Figure 1 .... **5**

Supplemental Figure 3 .... **7**

References 8

Experimental

Materials & Methods

Strains and colony biofilms culture conditions

Inocula for each microbial species were prepared independently for single-species and polymicrobial biofilm growth. SH1000 and PAO1-L strains were propagated in LB broth for 16 h at 37°C with 200 rpm shaking. C. albicans SC5314 was grown in yeast peptone dextrose (YPD [Oxiod, Cambridge, UK) for 16 h at 30°C in a shaking incubator at 200 rpm. Cells were washed (x3) in phosphate-buffered saline (PBS), and inocula for each specific microbe were prepared at 1x104, 1x106 and 1x105 CFU/mL for PAO1-L, SH1000 and SC5314, respectively. UVC-sterilised polycarbonate (PC) discs (0.2 µm pore size and 13 mm diameter, Sigma) deposited on wells of 6-well plates filled with 5 mL of Roswell Park Memorial Institute (RPMI) -1640 medium (without sodium bicarbonate and phenol red, Sigma-Aldrich) supplemented with 1.5% agar (w/v) and 165 mM MOPs (Sigma-Aldrich).

For treatment with the QS inhibitors, SEN19 and SEN89 compounds were supplemented at 10 µM in the final stock inoculum of PAO1-L. Treatment of 18 h PAO1-L colony biofilms with ciprofloxacin was performed by adding 20 µL of 64 µg/mL in H2O pipetted gently on top of the preformed colony biofilm. At endpoint, PC discs with attached colony biofilms were aseptically removed and agar plugs were used for QS signal detection. In parallel RPMI-1640 agar samples without microbial inoculation were used as background control for LESA mass spectrometry analysis. Triplicate biological and technical repeats were conducted for all experiments presented.

Colony-forming unit (CFU) counting[1]

PC discs were removed from agar and transferred to separate 1 mL microcentrifuge tubes containing 1 mL of 1x PBS and 5x 2.6 mm zirconium ceramic oxide beads (Fisherbrand, Loughborough, UK). The samples were then vortexed to remove the biofilm from the PC discs. The PC discs were removed and the biofilm disaggregated by bead beating for 30 sec using a FastPrep-24™ 5G Homogenizer (MP Biomedicals, Loughborough, UK). The 1 mL volume was then transferred to a 5 mL Bijou containing 4 mL 1x PBS. Bijous were then sonicated, in a sonicating water bath for 15 min at 37 kHz. Ten-fold serial dilutions were then performed in 1x PBS from the sonicated samples and 20 µL of each dilution were plated in triplicate on the appropriate selection agar. For monospecies PAO1-L = *Pseudomonas* isolation agar (PIA [Oxoid, Cambridge, UK]). Polymicrobial biofilms, PAO1-L = PIA + 4 µg/mL nystatin (Sigma-Aldrich, Gillingham, UK), *S. aureus* SH1000 = Mannitol salt agar ([Oxoid, Cambridge, UK]) + 4 µg/mL nystatin and *C. albicans* SC5314 = SAB + 125 µg/mL of tetracycline (Sigma-Aldrich, Gillingham, UK). Plates were incubated for 24 h at 37°C and colonies enumerated.

Pyocyanin assay

Pyocyanin colorimetric quantification assay was adapted from Essar and colleagues[2]. Briefly, PC discs with biofilms were removed and the agar was excised using a sterile scalpel and placed in a 15 mL falcon tube. To each falcon tube 7.5 mL of dH2O and 4.5 mL of Chloroform was added under fume cabinet and shaken vigorously, then centrifuged at 10,000 x g at 4°C for 10 min. The yellow top layer was then removed by pipette and 3 mL of the chloroform layer was transferred to a new falcon containing 1.5 mL 0.2M HCL and vortexed for 10 s. The falcons were then centrifuged at 10,000 x g at 4°C for 2 min. Avoiding transfer of residual chloroform, 1 mL of the pink top layer was transferred by pipette to cuvettes and the OD520 nm obtained. The pyocyanin concentration can then be determined by multiplying the OD520 nm value by 17.072 and then 1.5.

Mass Spectrometry

Tandem (MSMS) experiments were conducted via high-energy collision-induced dissociation (HCD). For MSMS experiments, AGC was used with a target of 1×10^6 charges and a maximum injection time of 500 ms. HCD was performed in the ion trap at a resolution of 70000 and a normalised collision energy between 20 and 25%, and fragments were detected in the Orbitrap. The isolation width was 1.0 Th.

Results

Quorum Sensing Molecules

For *P. aeruginosa* QS signal detection, we focused on two families of molecules. (i) The *N*-acyl homoserine lactones (AHLs), including *N*-(3-oxododecanoyl)-L-homoserine lactone (3-oxo-C12-HSL) and *N*-butanoyl-L-homoserine lactone (C4-HSL) produced and sensed by the LasRI and RhlRI systems, respectively[3]. (ii) The 2-alkyl-4(1*H*)-quinolones (AQs), including 2-heptyl-3-hydroxy-4(1H)-quinolone (PQS, pseudomonas quinolone signal) that can then promote the production of quinolones and virulence factors[4]. *P. aeruginosa* also produces AQs such as 2,4-dihydroxyquinoline (DHQ); synthesis of which was shown not to require oxygen and was found to sustain *P. aeruginosa* pyocyanin production and antifungal activity[5] and the AQNOs that do not function as QS signals. For example, 2-nonyl-4-hydroxyquinoline N-oxide (NQNO) and 2-heptyl-4-hydroxyquinoline N-oxide (HQNO) both effective anti-staphylococcal molecules[6, 7]. Moreover, HQNO is a potent cytochrome inhibitor for prokaryotic and eukaryotic cells[8]. The genes required for AQ biosynthesis (*pqsABCDE*) and response-regulation (*pqsR*) are located at the same genetic locus, although *pqsH* and *pqsL* are distally located[9].

MSMS

High-energy collision-induced dissociation (HCD) of m/z 244.17 confirmed that this is HHQ; the MSMS spectrum in Supplemental Figure 1A confirms fragments at m/z 159.07 and 172.08 that typify HHQ derived compounds[10, 11]. HCD fragmentation of m/z 260.16 shows characteristic fragments of HQNO at m/z 159.07, 172.08, and 186.09, alongside characteristic fragments of PQS at m/z 188.10, 175.06 and 162.06 in lower abundance confirming that both C7-PQS and C7-HQNO isomers are present in the sample, see Supplemental Figure 1B. Numerous C7 AQs have been shown to be elevated in virulent strains of *P. aeruginosa* previously[12]. Upon dissociation of m/z 272.20 the following product ions were detected; m/z 159.08, 172.07 however there was no fragment at m/z 188.10 or 175.06 suggesting that this species is NHQ rather than C9-PQS.

Supplemental Figures

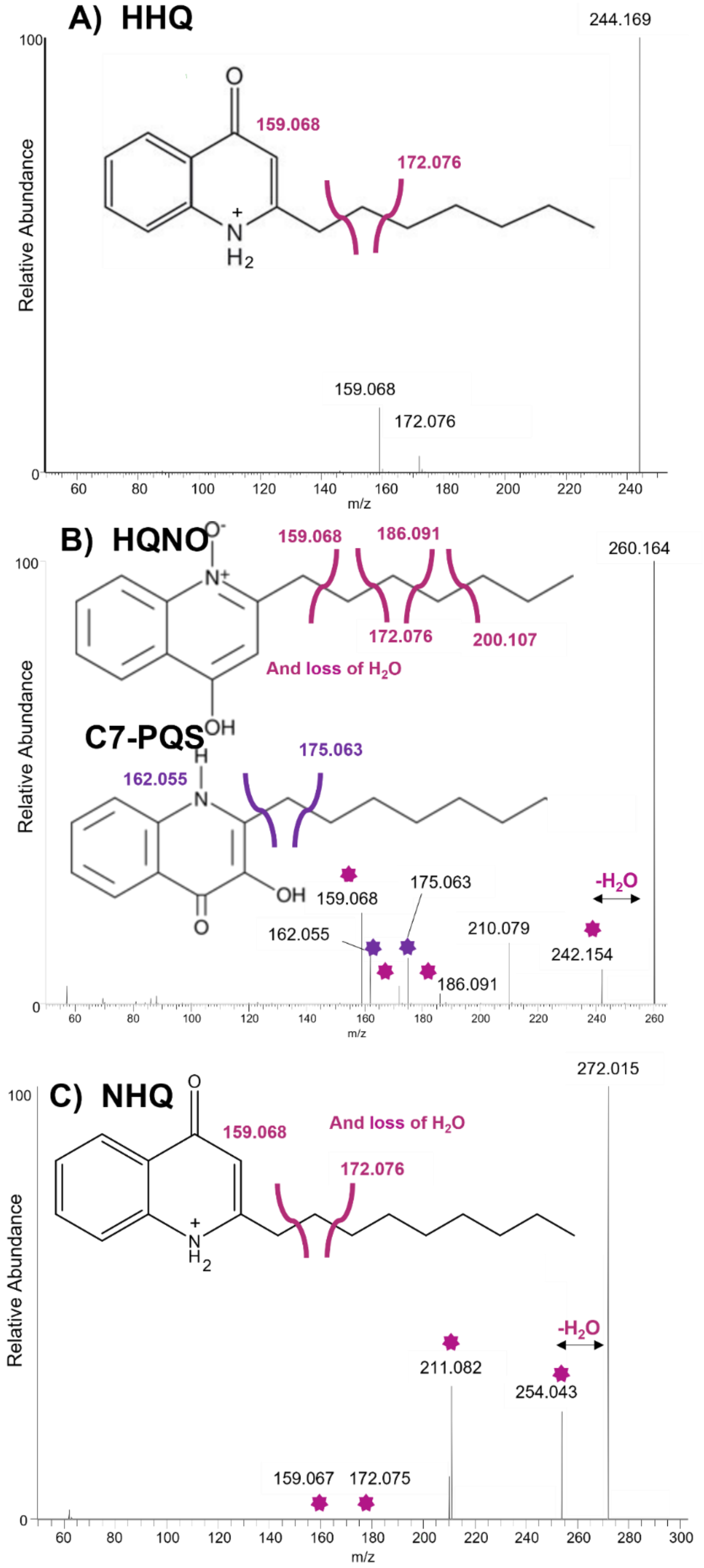

Supplemental Figure 1 LESA MS/MS of A) m/z 244.169, B) m/z 260.164 and C) m/z 272.015.

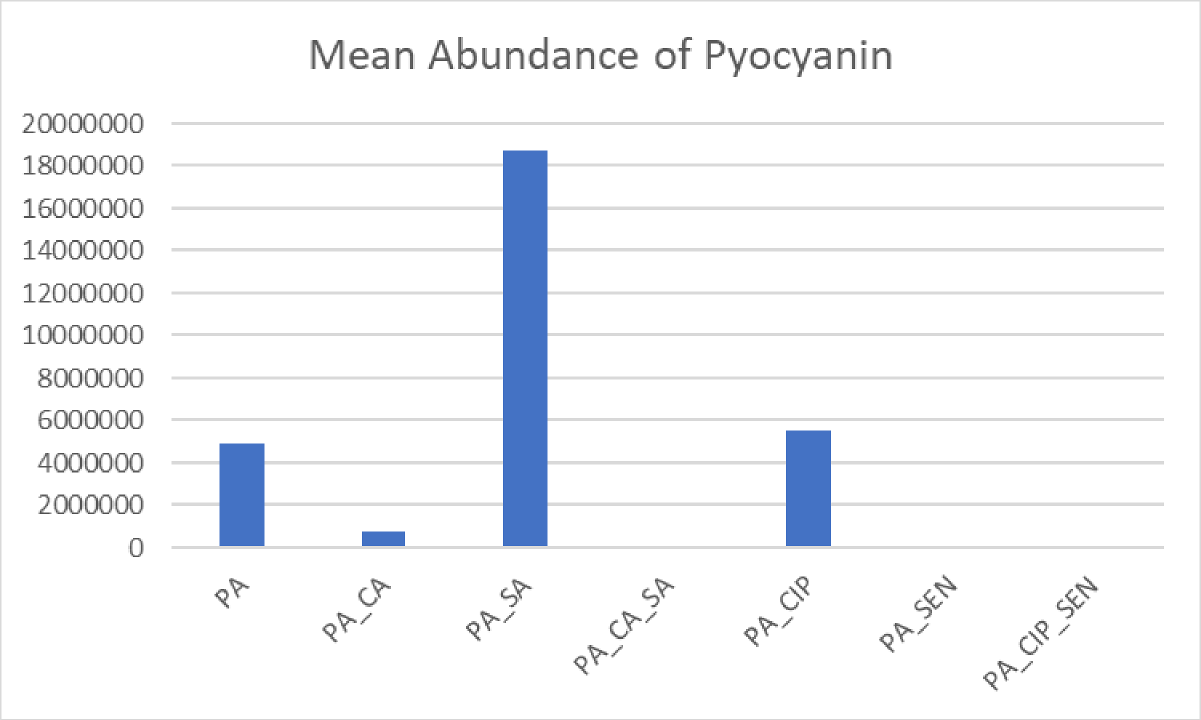

Supplemental Figure 2 Bar chart showing pyocyanin abundance in LESA-MS experiments of *Psuedomonas aeruginosa* (PA), drug or inhibitor treated PA, and PA in the presence of Staphylococcus aureus (SA) or Candida albicans (CA). Pyocyanin was detected in higher abundance in exo-metabolome analysis in the presence of *S. aureus*, and reduced in the presence of *C. albicans*. Production was unaffected by antibiotic treatment with ciprofloxacin (CIP) and was inhibited by the quorum sensing inhibitors (SEN) studied.

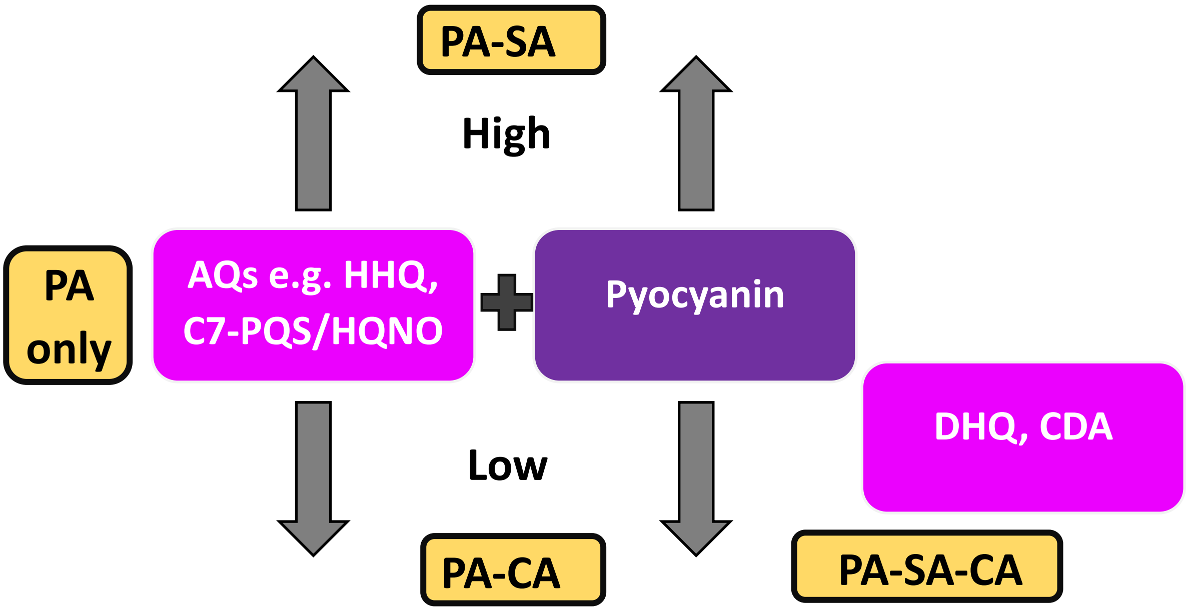

Supplemental Figure 3 Summary of diagnostic analytes detected in the exo-metabolome of interspecies biofilms via LESA-MS

References

1. Miles, A. A.; Misra, S. S.; Irwin, J. O., The estimation of the bactericidal power of the blood. *J Hyg (Lond)* **1938,** *38* (6), 732-49.
2. Essar, D. W.; Eberly, L.; Hadero, A.; Crawford, I. P., Identification and characterization of genes for a second anthranilate synthase in Pseudomonas aeruginosa: interchangeability of the two anthranilate synthases and evolutionary implications. *J Bacteriol* **1990,** *172* (2), 884-900.
3. Williams, P.; Cámara, M., Quorum sensing and environmental adaptation in Pseudomonas aeruginosa: a tale of regulatory networks and multifunctional signal molecules. *Current Opinion in Microbiology* **2009,** *12* (2), 182-191.
4. Ilangovan, A.; Fletcher, M.; Rampioni, G.; Pustelny, C.; Rumbaugh, K.; Heeb, S.; Cámara, M.; Truman, A.; Chhabra, S. R.; Emsley, J.; Williams, P., Structural basis for native agonist and synthetic inhibitor recognition by the Pseudomonas aeruginosa quorum sensing regulator PqsR (MvfR). *PLoS Pathog* **2013,** *9* (7), e1003508.
5. Rella, A.; Yang, M. W.; Gruber, J.; Montagna, M. T.; Luberto, C.; Zhang, Y.-M.; Del Poeta, M., Pseudomonas aeruginosa Inhibits the Growth of Cryptococcus Species. *Mycopathologia* **2012,** *173* (5), 451-461.
6. Hoffman, L. R.; Déziel, E.; D'Argenio, D. A.; Lépine, F.; Emerson, J.; McNamara, S.; Gibson, R. L.; Ramsey, B. W.; Miller, S. I., Selection for <i>Staphylococcus aureus</i> small-colony variants due to growth in the presence of <i>Pseudomonas aeruginosa</i>. *Proceedings of the National Academy of Sciences* **2006,** *103* (52), 19890-19895.
7. Szamosvári, D.; Böttcher, T., An Unsaturated Quinolone N-Oxide of Pseudomonas aeruginosa Modulates Growth and Virulence of Staphylococcus aureus. *Angewandte Chemie International Edition* **2017,** *56* (25), 7271-7275.
8. Heeb, S.; Fletcher, M. P.; Chhabra, S. R.; Diggle, S. P.; Williams, P.; Cámara, M., Quinolones: from antibiotics to autoinducers. *FEMS Microbiology Reviews* **2011,** *35* (2), 247-274.
9. Winsor, G. L.; Lam, D. K. W.; Fleming, L.; Lo, R.; Whiteside, M. D.; Yu, N. Y.; Hancock, R. E. W.; Brinkman, F. S. L., Pseudomonas Genome Database: improved comparative analysis and population genomics capability for Pseudomonas genomes. *Nucleic Acids Res* **2010,** *39* (suppl_1), D596-D600.
10. Lépine, F.; Milot, S.; Déziel, E.; He, J.; Rahme, L. G., Electrospray/mass spectrometric identification and analysis of 4-hydroxy-2-alkylquinolines (HAQs) produced by Pseudomonas aeruginosa. *Journal of the American Society for Mass Spectrometry* **2004,** *15* (6), 862-869.
11. Lanni, E. J.; Masyuko, R. N.; Driscoll, C. M.; Dunham, S. J. B.; Shrout, J. D.; Bohn, P. W.; Sweedler, J. V., Correlated Imaging with C60-SIMS and Confocal Raman Microscopy: Visualization of Cell-Scale Molecular Distributions in Bacterial Biofilms. *Analytical Chemistry* **2014,** *86* (21), 10885-10891.
12. Depke, T.; Thöming, J. G.; Kordes, A.; Häussler, S.; Brönstrup, M., Untargeted LC-MS Metabolomics Differentiates Between Virulent and Avirulent Clinical Strains of Pseudomonas aeruginosa. *Biomolecules* **2020,** *10* (7), 1041.
